# A randomized, double-blind, single-dose study to evaluate the biosimilarity of QL1101 with bevacizumab in healthy male subjects

**DOI:** 10.1007/s00280-019-04014-x

**Published:** 2020-01-06

**Authors:** Ya-nan Liu, Jie Huang, Can Guo, Shuang Yang, Ling Ye, Shu-ting Wu, Xing-fei Zhang, Xiao-yan Yang, Cui-cui Han, Qi Pei, Lu Huang, Qing-nan He, Guo-ping Yang

**Affiliations:** 1 Clinical Trails Center of the Third Xiangya Hospital, Central South University, Changsha, Hunan China; 2 XiangYa School of Pharmaceutical Sciences, Central South University, Changsha, Hunan China; 3 Qilu Pharmaceutical Co. Ltd, Jinan, Shangdong China; 4 Department of Pharmacy of the Third Xiangya Hospital, Central South University, Changsha, Hunan China; 5 Research Center for Drug Clinical Evaluation, Central South University, Changsha, Hunan China; 6 Department of Pediatrics of the Third Xiangya Hospital, Central South University, Changsha, Hunan China; 7 Center for Clinical Pharmacology of the Third Xiangya Hospital, Central South University, Changsha, Hunan China

**Keywords:** QL1101, Bevacizumab, Biosimilar, Pharmacokinetics, Safety, Immunogenicity

## Abstract

**Purpose:**

This is the first study to compare the pharmacokinetics of QL1101, a proposed bevacizumab biosimilar, with Avastin^®^ sourced from Roche Diagnostics GmbH.

**Methods:**

In this double-blind, single-dose, parallel-group study, healthy male subjects were randomized 1:1 to receive QL1101 or Avastin^®^ 3 mg/kg intravenously. Pharmacokinetic assessments were conducted for 85 days, with additional safety and immunogenicity assessments until day 90. Primary study endpoints were area under the concentration–time curve (AUC) from time zero to infinity (AUC_0–∞_), AUC from time zero to the last quantifiable concentration (AUC_0–last_), and maximum serum concentration (*C*_max_). Pharmacokinetic equivalence was shown if the 90% confidence intervals (CIs) of the geometric mean ratios (GMRs) of the *C*_0–max_, AUC_0–last_, and AUC_0–∞_ were within the predefined bioequivalence margin of 80–125.00%.

**Results:**

A total of 82 subjects were randomized to the following groups: 42 to QL1101 and 40 to Avastin^®^. The 90% CIs of the GMRs of AUC_0–∞_, AUC_0–last_, and C_max_ of QL1101 and Avastin® were (97.8%, 107.0%), (94.5%, 106.9%), and (94.1%, 107.3%), respectively, which were all within the bioequivalence margin. The incidence of adverse events was 90.5% and 95.0% in the QL1101 and Avastin® groups, respectively. Mean serum concentration–time profiles, secondary pharmacokinetic parameters, and safety and immunogenicity profiles were comparable across the two treatment groups.

**Conclusions:**

The study demonstrated the pharmacokinetic equivalence of QL1101 to Avastin^®^. QL1101 (3 mg/kg, iv) is safe and tolerable in healthy Chinese subjects. These data support the further clinical evaluation of QL1101 as a bevacizumab biosimilar.

## Introduction

Monoclonal antibodies (mAbs) targeted to critical pathways involved in cancer pathogenesis and growth factors are often used to reduce or ameliorate treatment-related hematological toxicity [1]. Recombinant monoclonal antibodies play an important role in the treatment of cancer [2, 3], and there is an urgent clinical demand. There is no doubt that biologics are one of the most promising and fastest-growing markets in the pharmaceutical industry [2].

A biosimilar is a biological product that is highly similar, but not identical, to a licensed biological product (the reference or originator product) [2]. The United States Food and Drug Administration (FDA), the World Health Organization (WHO), and European Medicines Agency (EMA) emphasized a stepwise approach for the demonstration of potential clinical efficacy of biosimilars [4–6]. According to guidelines from the FDA, the scope of the clinical program and the type of clinical studies (i.e., comparative human pharmacokinetics, pharmacodynamics, clinical immunogenicity, or clinical safety and effectiveness) should be scientifically justified by the sponsors [4–6]. In this study, as the first step to demonstrate the biosimilarity of QL1101, we compared this proposed bevacizumab biosimilar with a reference product in the assessment of human pharmacokinetics.

Angiogenesis plays a central role in tumor growth, invasion, and metastasis. Vascular endothelial growth factor (VEGF) is an important factor in angiogenesis. After VEGF binds to receptors on vascular epithelial cells, it promotes the amplification, migration, microtubule formation, and microvascular permeability of vascular epithelial cells through the intercellular signal transduction system, and thus induces angiogenesis to support tumor growth [7, 8]. The humanized monoclonal antibody bevacizumab (Avastin^®^) is an inhibitor of VEGF and is indicated for the treatment of an array of tumor types [9], including metastatic colorectal cancer [10], metastatic breast cancer [11], non-small-cell lung cancer [12], and metastatic renal cell carcinoma [13]. Bevacizumab (Avastin^®^) was first approved by the FDA in February 2004 as the first targeted drug of VEGF for chemotherapy combined with 5-fluorouracil. This combination was applied to metastatic colon or rectal cancer as a first-line therapy [14]. In addition, in January 2005, bevacizumab was approved in the EU [15] and later approved in China in February 2010. The first bevacizumab biosimilar (Mvasi^®^), developed by Amgen and Allergan, was approved by the FDA in September 2017 [16], and later approved in the EU in January 2018 [17].

The recombinant anti-VEGF humanized monoclonal antibody injection (QL1101), developed by Qilu Pharmaceutical Co. Ltd, has completed the production process research, quality research, and preclinical pharmacological toxicology research (data not shown). QL1101 is expressed in Chinese hamster ovary (CHO) cell lines and the manufacturing process consists of cell culture, harvest, and purification steps. The preclinical safety, effectiveness, and pharmacokinetic behaviors of the products are similar to that of the bevacizumab reference product. Therefore, these results have supported the clinical development of bevacizumab biosimilars.

The primary objective of this study was to demonstrate the pharmacokinetic equivalence of QL1101 versus that of the reference product bevacizumab sourced from Roche Diagnostics GmbH. The secondary objectives were to assess safety and immunogenicity. To the best of our knowledge, this is the first clinical study comparing the pharmacokinetic properties of QL1101 to bevacizumab to investigate its potential as a bevacizumab biosimilar.

## Methods

### Statement of human rights

The final protocol, any amendments, and informed consent documentation were reviewed and approved by the Institutional Review Board of the Third Xiangya Hospital of Central South University. The study was conducted in compliance with the Declaration of Helsinki, International Conference on Harmonization Good Clinical Practice Guidelines, and local regulatory requirements. All subjects gave their informed consent before their inclusion in this study.

### Study population

Healthy subjects have no underlying diseases or concomitant drug use, and PK parameters were more consistent among individuals. Considering that the study may have an impact on female fertility, so healthy male subjects are planned to be included in this study. The healthy male volunteers aged 18–40 years, with body mass index ranging between 19.0 and 26.0 kg/m^2^ and a body weight ≥ 50 kg, were enrolled in the study. At the time of enrollment, all subjects had normal organ function evaluated by laboratory analysis. Subjects with evidence or history of clinically significant diseases, previous history of cancer, hypertension (defined as systolic blood pressure > 140 or < 90 mmHg), or heart rate > 100 bpm or < 50 bpm were excluded from the study. Subjects were excluded if they had received blood transfusions, previous anti-VEGF treatment with antibodies or proteins, or were positive for the anti-VEGF antibody.

### Study design

This study was a single center, randomized, double-blind, single-dose, parallel controlled study that was conducted in the Phase I Clinical Research Center of the Third Xiangya Hospital of Central South University (Clinical trial registration number is ChiCTR1900022767).

After the screening (7 days prior to drug administration), the eligible subjects were admitted to the Clinical Research Center 1 day before dosing. All subjects fasted for at least 8 h before dosing and were then divided into two groups with a 1:1 ratio: one group was administrated with a single dose of QL1101 3 mg/kg and the second group was administered Avastin^®^ 3 mg/kg according to a computer-generated randomization schedule. Subjects were administrated with a single dose of allocated study drug (diluted in 0.9% sodium chloride solution) as an intravenous infusion over 90 min.

### Pharmacokinetic evaluations

Blood samples for pharmacokinetic (PK) evaluation were collected at 0 h before the initiation of dosing (pre-dose), and at 45 min, 90 min, 2, 4, 8, 12, 24, 48, 96, 168, 336, 504, 672, 840, 1008, 1344, and 1680 h after drug infusion. Blood samples were allowed to clot for 30 min at room temperature and centrifuged at 3000 rpm for 15 min at 2–8 °C. The serum was stored at − 80 °C for further analysis.

The concentrations of the bevacizumab biosimilar (QL1101) and bevacizumab in the serum were analyzed by enzyme-linked immunosorbent assay (ELISA) using plates coated with recombinant human VEGF at the WuXi Apptec Co. Ltd. (Shanghai, China). Analytical determination method linear range was 31.25–2000.0 ng/mL. The precision range of quality control samples was 5.2–9.5%CV and the accuracy deviation range of each quality control sample was − 6.4–5.7%.

### Immunogenicity evaluations

Blood samples for detecting anti-drug antibodies (ADAs) and neutralizing antibodies (NABs) were collected before the start of study drug infusion on day 1 and on days 15, 43, and 85 post-dose. Subjects were followed up for 3 months after the end of the study if they tested positive for ADAs, if they tested negative for bevacizumab in two consecutive blood samples (whichever happened first), or if acceptable stable status was achieved by the investigators and the sponsors. ADAs samples were analyzed at the WuXi Apptec Co. Ltd. (Shanghai, China) using the validated electrochemiluminescent assays to detect QL1101 and Avastin^®^. Samples with ADA positivity were further tested for the presence or absence of neutralizing anti-bevacizumab biosimilars or anti-bevacizumab antibodies, using validated electrochemiluminescent NABs assays.

### Safety evaluations

Safety was evaluated by vital signs, physical examination, ECG, laboratory examination (blood routine, blood biochemistry, blood coagulation function, urine routine, fecal routine, fecal occult blood, and blood transfusion), adverse events (AEs), and combined medication. AEs were recorded and graded according to the National Cancer Institute Common Terminology Criteria for Adverse Events (version 4.03). All AEs were assessed and scored based on their severity and relation to bevacizumab and its biosimilars. Subjects with AEs were monitored until the condition was resolved or stabilized.

### Statistical analyses

The calculated PK parameters using standard non-compartmental analysis of concentration–time data included maximum observed serum concentration (*C*_max_), AUC from zero to the time of the last quantifiable concentration (AUC_0–last_), AUC from zero extrapolated to infinity (AUC_0–∞_), clearance (CL), apparent volume of distribution (*V*_z_), and terminal half-life (*t*_1/2_). PK parameters were calculated using an internally validated software system, Phoenix WinNonLin^®^ version 6.3 (Certara US, Inc., Princeton, NJ, USA).

Pharmacokinetic bioequivalence between QL1101 and Avastin^®^ was present if the 90% confidence intervals (CIs) for *C*_max_, AUC_0–last_, and AUC_0–∞_ were between 80 and 125.00%. The analysis was conducted using an analysis of variance (ANOVA) model on the logarithmic scale, and then, the bioequivalence between the drugs was evaluated and judged using the two one-sided *t* test or Wilcoxon rank tests. All statistical tests were performed by SAS Version 9.3 (SAS Institute Inc., Cary, NC, USA), and *P* < 0.05 was considered statistically significant.

Analyses of safety data were conducted in the safety population and AEs were listed by subjects’ test number (Medical Dictionary for Regulatory Activities [MedDRA], version 20.0). All safety and immunogenicity data were analyzed descriptively.

## Results

### Demographic and baseline characteristics

**Fig. 1:**
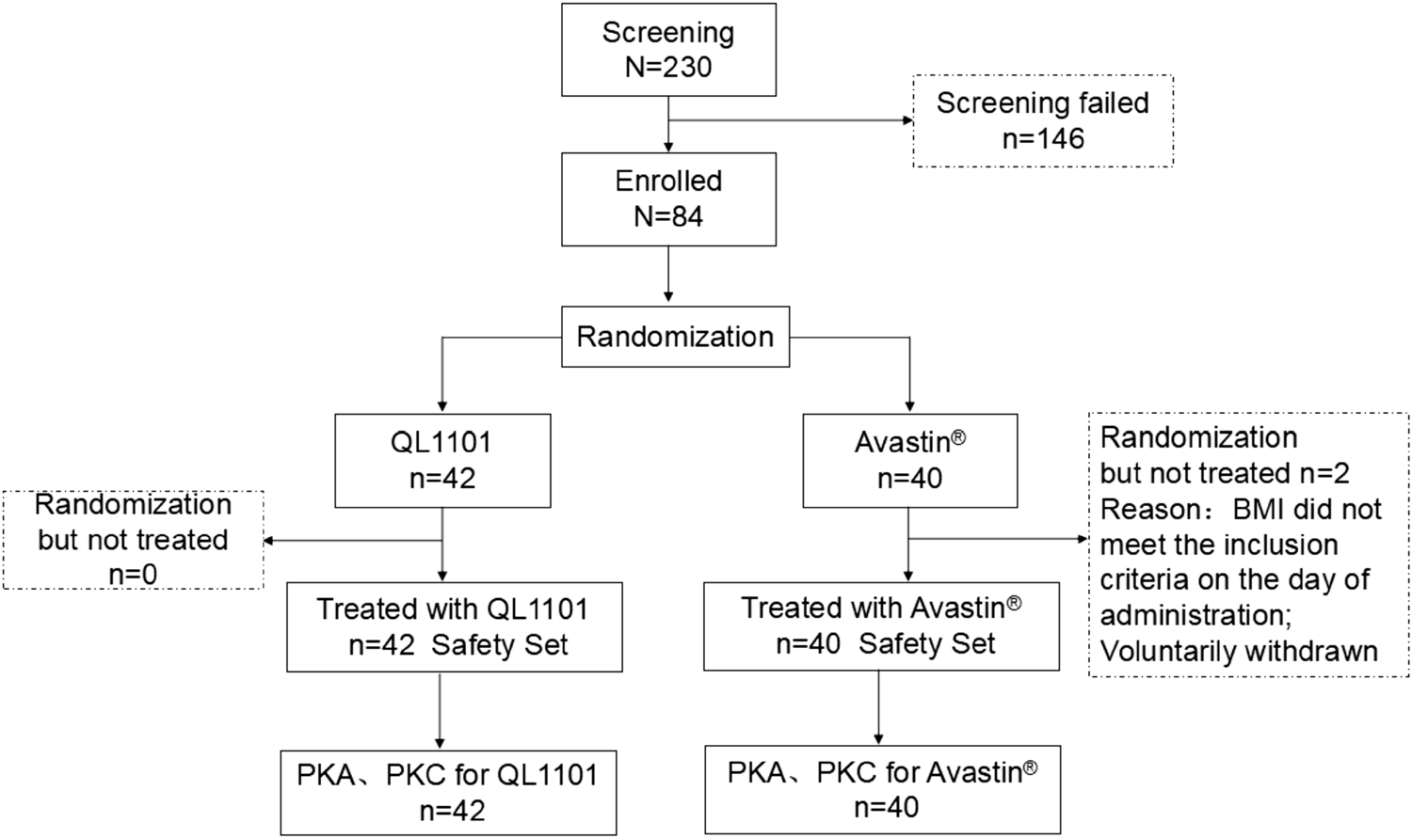
Subject disposition. *BMI* body mass index, *PKA* pharmacokinetic analytic set, *PKC* pharmacokinetic concentration detection set

This study was conducted between 24 March 2016 and 9 October 2017. A total of 84 subjects were enrolled and assigned in the study, though 82 received the assigned study drug (QL1101, *n* = 42; Avastin^®^, *n* = 40) and constituted the safety analysis set (Fig. 1). Two subjects withdrew; one withdrew, since their BMI did not meet the inclusion criteria on the day of administration and one voluntarily withdrew. Both were from the Avastin^®^ group. The final per-protocol population used in the PK analysis consisted of 42 and 40 subjects in the QL1101 and Avastin^®^ groups, respectively (Fig. 1). The demographic and baseline characteristics of all subjects are presented in Table 1. There were no significant differences between the demographic and baseline parameters among the two groups.

**Table 1:** Demographic and baseline characteristics of health male subjects

	QL1101 (*n* = 42)	Avastin^®^ (*n* = 40)
Age (years)		
Mean (± SD)	25.6 (± 4.7)	24.9 (± 3.5)
Range	18–36	19–32
Weight (kg)		
Mean (± SD)	64.0 (± 6.9)	64.1 (± 7.0)
Range	52.6–82	51–76.4
Height (cm)		
Mean (± SD)	168.7 (± 6.0)	169.5 (± 5.0)
Range	153.0–186.0	160.0–181.0
Body mass index (kg/m^2^)		
Mean (± SD)	22.5 (± 2.0)	22.2 (± 1.7)
Range	19.4–25.7	19.1–25.6
Ethnicity (Han/other)	41/1	38/2

The data are Mean (± SD) or Range (minimum–maximum)

*SD* standard deviation

### Pharmacokinetic evaluations

**Fig. 2:**
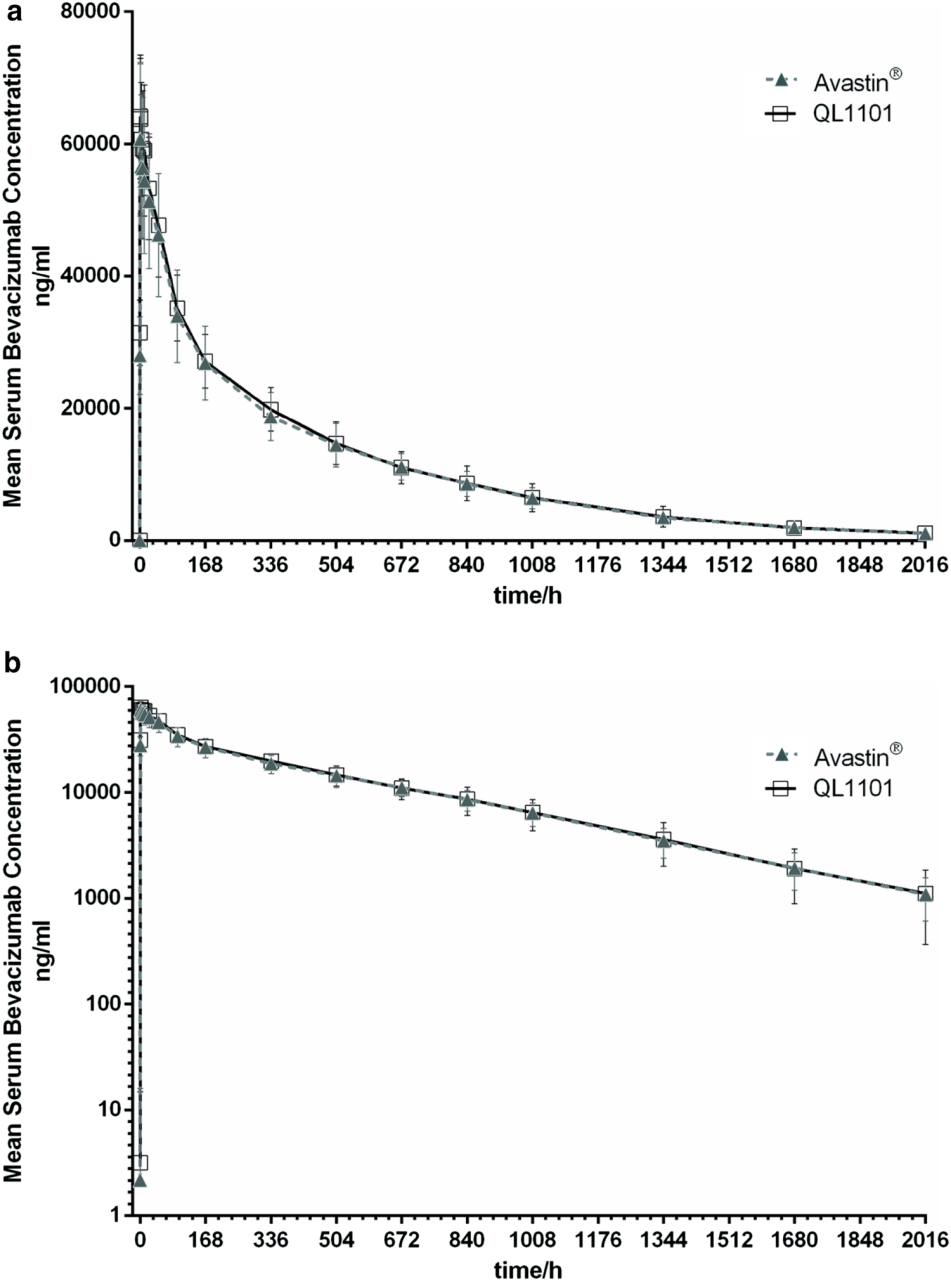
**a** Mean serum concentration–time profiles following a single 3 mg/kg intravenous dose in healthy subjects of a linear scale. **b** Mean serum concentration–time profiles following a single 3 mg/kg intravenous dose in healthy subjects of a semi-logarithmic scale

The mean serum concentration–time profiles of the two study drugs are shown in Fig. 2, which exhibited a similar trend. *C*_max_, AUC_0–last_, and AUC_0–∞_, and the secondary pharmacokinetic endpoints (*T*_max_, *V*_z_, *T*_1/2_, and CL) were similar in the two groups (Table 2). Treatment group comparison (QL1101/Avastin^®^) and the 90% CIs of the GMR for QL1101/Avastin^®^ of *C*_max_, AUC_0–last_, and AUC_0–∞_ were all within the predefined bioequivalence margin 80–125.00% (Table 3).

**Table 2:** Pharmacokinetic parameters of QL1101 and Avstain^®^

Parameters (units)	QL1101 (*n* = 42)	Avastin^®^ (*n* = 40)
*T*_max_^a^ (h)	3.46 (1.43,13.65)	3.49 (1.40,13.63)
*C*_max_^b^ (ng/mL)	66001.30 ± 9003.50	64337.79 ± 7246.91
AUC_0-last_^b^ (h*ng/mL)	21628158.58 ± 4291590.17	21366803.97 ± 3,082,243.33
AUC_0-inf_^b^ (h*ng/mL)	22304035.24 ± 4792983.85	22004023.81 ± 3332419.46
*T*_1/2_^b^ (h)	378.51 ± 75.57	384.90 ± 56.56
*V*_z_^b^ (mL)	4707.91 ± 799.33	4855.16 ± 768.13
CL^b^ (mL/h)	8.86 ± 1.78	8.86 ± 1.54

*AUC*_*0–∞*_ area under the serum concentration–time curve from zero extrapolated to infinity, *AUC*_*0–last*_ area under the serum concentration–time curve from zero to the time of the last quantifiable concentration, *CL* clearance, *C*_*max*_ maximum observed serum concentration, *T*_*1/2*_ terminal half-life, *V*_*ss*_ volume of distribution at steady state

^a^*T*_max_ is represented by the median (minimum, maximum)

^b^*C*_max_, AUC_0-last_, AUC_0-inf_, *T*_1/2_, *V*_z_, and CL are represented by the mean ± standard deviation

**Table 3:** Statistical comparison of pharmacokinetic parameters

Parameters (units)	Geometric means and ratio% (90% CI)		
	QL1101 (*n* = 42)	Avastin^®^ (*n* = 40)	Ratio % (90% CI)^a^
*C*_max_ (ng/mL)	65418.81	63950.39	102.30% (97.80–107.00)
AUC_0–last_ (h*ng/mL)	21251848.70	21145370.92	100.50% (94.50–106.90)
AUC_0-inf_ (h*ng/mL)	21855737.07	21752228.39	100.50% (94.10–107.30)

^a^Ratio(%) is the geometric means ratio of QL1101 to Avastin^®^

### Immunogenicity evaluations

Of the 82 subjects in the safety population, all completed ADAs and NABs at specified visit days. In the QL1101 group, three subjects tested positive for ADAs and NABs on day 85. At the other follow-up visits (about 3 months), three subjects still tested positive for ADAs and 2 were positive for NABs. The medical history and safety evaluations were conducted for 3 subjects with ADA positivity. None of the three subjects had serious adverse reactions during the study, and none had significant and clinically significant changes in laboratory test values. In the Avastin^®^ group, all subjects were negative for ADAs and NABs. Due to limited scientific approach, only a simple statistical description was carried out. Therefore, the comparison of immunogenicity needs to be tested in a larger sample size study in the future.

### Safety evaluations

No deaths or discontinuations due to adverse events (AEs) occurred in this study. A total of 76 (92.7%) subjects in the safety population reported one or more AEs during the study (90.5% and 95.0% in the QL1101 and Avastin^®^ groups, respectively), though there was no statistically significant difference between the two groups. Among the 82 subjects who received QL1101 and Avastin^®^, 34 and 30 experienced adverse reactions (ARs) with the incidence of 81.0% and 75.0%, respectively. There was no statistically significant difference between the two groups. There were two severe adverse events (SAEs) in two subjects: 1 in the QL1101 group, which was definitely unrelated to the study drug group, and 1 in the Avastin^®^ treatment group which was unrelated to the study drug. SAE incidence was 2.4% and 2.5%, respectively, for the QL1101 and Avastin^®^ groups and there was no statistically significant difference between the two groups. There were 23 (25 cases) of major adverse events (MAEs) in the subjects: 14 cases (15 cases) and 9 cases (10 cases) in the QL1101 treatment group and in the Avastin^®^ treatment group, respectively, with an incidence rate of 33.3% and 22.5%, respectively. However, there was no statistically significant difference between the two groups.

Laboratory results, physical examination findings, vital signs, and electrocardiogram values were unremarkable, with no safety issues identified and with no clinically meaningful differences among the two treatment groups. In total, there were no statistically significant differences between the two groups with regard to adverse events, adverse reactions, serious adverse events, and important adverse events. The results are shown in Table 4.

**Table 4:** Summary of adverse events by category

Adverse events [*n *(%)]	QL1101 (*n* = 42)	Avastin^®^ (*n* = 40)
Subjects with AEs	38 (90.5)	38 (95.0)
Subjects with ARs	34 (81.0)	30 (75.0)
Subjects with SAEs	1 (2.4)	1 (2.5)
Subjects with SARs	0	0
Subjects with MAEs	14 (33.3)	9 (22.5)
Most common AEs^a^		
Upper respiratory infection	13 (40.0)	7 (17.5)
Blood triglyceride increased	12 (28.6)	12 (30.0)
Conjugated bilirubin increased	7 (16.7)	4 (10.0)
Blood bilirubin increased	6 (14.3)	4 (10.0)
Total bile acid increased	6 (14.3)	4 (10.0)
Abdominal pain	5 (11.9)	4 (10.0)
Diarrhea	5 (11.9)	5 (12.5)

*AEs* adverse events, *MedDRA* Medical Dictionary for Regulatory Activities, version 20.0, *ARs* adverse reactions, *SAEs* serious adverse events, *SARs* serious adverse reactions, *MAEs* major adverse events

^a^Most common AEs, AEs reported by ≥ 10% subjects in any treatment group

## Discussion

The market for biosimilars still is not established and their ability to penetrate clinical practice still is not confirm, so more and more countries are taking steps on paving the way for biosimilars. In 2018, the FDA’s announced Biosimilars Action Plan will take steps to increase access for patients who need biologic medication to biosimilar drugs that are nearly identical to but potentially much less expensive than the original product [18]. Additionally, some experts suggest that investigating the efficacy of a biosimilar in extended indications may be a way for manufacturers of biosimilar agents to leverage additional value over the reference biologic agent [19].

The biosimilars of bevacizumab are not marketed in China. QL1101 is one of the fastest-developing bevacizumab biosimilars in China. In this study, we referred to the research design of similar products abroad, such as PF-06439535 [20], BI-695502 [21], ABP-215 [22] and so on, in which ABP-215 (Mvasi^®^) has been approved by the FDA and the EU. According to previous data [20–23], we assumed that the coefficient of intra-individual variation was 25%. If the geometric mean ratio (GMR) was set to be 95–105% to achieve 90% power (1-β) at the 5% nominal level (*α* = 5%), 37 evaluable subjects were required to be in each treatment group to meet the bioequivalence in the range of 80–125.00%. Considering the 10% drop-out rate and random grouping, the final total sample group size was 84 (42 subjects per arm).

The linear dosage range of the original research product Avastin^®^ is 1–10 mg/kg [24]. In this study, 3 mg/kg was selected as the low dose in the linear dose range, for safety purposes. This dose is less than the clinical dose for colorectal cancer and a fifth of the dose used for non-small cell lung cancer. Therefore, we hypothesized that this dose could minimize the potential damage to healthy subjects caused by the study drug. The single dose of ABP-215 used in healthy people was 3 mg/kg and that of PF-06439535 was 5 mg/kg, which demonstrated the safety of these doses in healthy male subjects. Several studies also chose safer doses for different compounds, such as BI695502 and DRL_B2 [25], which were infused at 1 mg/kg for 30 min. Although the dose and biological sample detection method are different, most of these studies have the same conclusion, which show that the biosimilars are equivalent to the original drug in terms of PK properties and safety evaluation.

The primary objective of the current phase I study was to demonstrate PK similarity of QL1101 to Avastin^®^ in healthy volunteers. The results revealed that the bevacizumab biosimilar (QL1101) has similar PK profiles to Avastin^®^ when administered in healthy male volunteers. The 90% CIs of the QL1101 to Avastin^®^ ratios for *C*_max_ and AUC were within the predefined bioequivalence acceptance range of 80–125.00%. And the follow-up study will approve primarily biosimilars on PD endpoints, such as the efficacy, safety, and immunogenicity.

This study was a single-dose study, which conducted in the healthy Chinese population, and the baseline differences of all subjects were not statistically significant between both groups. There was no significant differences in adverse events, laboratory results, or the evaluation of biosimilarity. The most common adverse events reported from bevacizumab treatment are hypertension, fatigue, diarrhea, and abdominal pain. In this study, the most common adverse events were upper respiratory infection, increased blood triglyceride, increased conjugated bilirubin, increased blood bilirubin, and increased total bile acid. Since the safety data reported in previous studies were evaluated for continuous administration in tumor patients, the adverse reactions were slightly different from the low-dose study design of healthy male subjects in this study. The comparison of immunogenicity needs to be tested in a larger sample size study in the future.

## Conclusion

The results of this Phase I study demonstrate that PK equivalence of QL1101 and Avastin^®^. QL1101(3 mg/kg, iv) is safe and tolerable in healthy Chinese subjects. QL1101 and Avastin^®^ were assessed in a Phase III study in patients with non-small cell lung cancer and the primarily purpose is to compare the efficacy and safety. The results of the Phase III study will be reported in a separate communication that is currently in development.

## References

[1] Rugo HS, Linton KM, Cervi P, Rosenberg JA, Jacobs I (2016). A clinician's guide to biosimilars in oncology. Cancer Treat Rev.

[2] Rodgers KR, Chou RC (2016). Therapeutic monoclonal antibodies and derivatives: historical perspectives and future directions. Biotechnol Adv.

[3] Santos SB, Sousa LJ, Silva AC (2019). Biosimilar medicines used for cancer therapy in Europe: a review. Drug Discov Today.

[4] European Medicines Agency (2014) Guideline on similar biological medicinal products. https://www.ema.europa.eu/docs/en_GB/document_library/Scientific_guideline/2014/10/WC500176768.pdf

[5] US Food and Drug Administration (2015) Scientific considerations in demonstrating biosimilarity to a reference product. https://www.fda.gov/downloads/drugs/guidance_compliance_regulatory_in_formation/guidances/ucm291128.pdf

[6] World Health Organization, Expert Committee on Biological Standardization (2009) Guidelines on evaluation of similar biotherapeutic products (SBPs). https://www.who.int/biologicals/areas/biological_therapeutics/BIOTHERAPEUTICS_FOR_WEB_22APRIL2010.pdf

[7] Ferrara N (2004). Vascular endothelial growth factor: basic science and clinical progress. Endocr Rev.

[8] Hicklin DJ, Ellis LM (2005). Role of the vascular endothelial growth factor pathway in tumor growth and angiogenesis. J Clin Oncol.

[9] Ferrara N, Hillan KJ, Gerber HP, Novotny W (2004). Discovery and development of bevacizumab, an anti-VEGF antibody for treating cancer. Nat Rev Drug Discov.

[10] Venook AP, Niedzwiecki D, Lenz HJ, Innocenti F, Fruth B, Meyerhardt JA, Schrag D, Greene C, O'Neil BH, Atkins JN, Berry S, Polite BN, O'Reilly EM, Goldberg RM, Hochster HS, Schilsky RL, Bertagnolli MM, El-Khoueiry AB, Watson P, Benson AR, Mulkerin DL, Mayer RJ, Blanke C (2017). Effect of first-line chemotherapy combined with cetuximab or bevacizumab on overall survival in patients with KRAS wild-type advanced or metastatic colorectal cancer: a randomized clinical trial. JAMA.

[11] Brufsky A (2016). Is there room for bevacizumab in metastatic breast cancer?. Lancet Oncol.

[12] Weiss J (2017). Bevacizumab in adjuvant treatment of non-small-cell lung cancer. Lancet Oncol.

[13] Hsieh JJ, Purdue MP, Signoretti S, Swanton C, Albiges L, Schmidinger M, Heng DY, Larkin J, Ficarra V (2017). Renal cell carcinoma. Nat Rev Dis Primers.

[14] US Food and Drug Administration. Original Approvals or Tentative Approvals (2004) https://www.accessdata.fda.gov/scripts/cder/daf/index.cfm?event=overview.process&ApplNo=125085

[15] European Medicines Agency. Avastin (bevacizumab): EPAR procedural steps taken before authorization (2005) https://www.ema.europa.eu/ema/index.jsp?curl=pages/medicines/human/medicines/000582/human_med_000663.jsp&mid=WC0b01ac058001d124

[16] Casak SJ, Lemery SJ, Chung J, Fuchs C, Schrieber SJ, Chow ECY, Yuan W, Rodriguez L, Gwise T, Rowzee A, Lim S, Keegan P, McKee AE, Pazdur R (2018). FDA's approval of the first biosimilar to bevacizumab. Clin Cancer Res.

[17] Melosky B, Reardon DA, Nixon AB, Subramanian J, Bair AH, Jacobs I (2018). Bevacizumab biosimilars: scientific justification for extrapolation of indications. Future Oncol.

[18] Voelker R (2018). Paving the way for biosimilars. JAMA.

[19] Tang GY (2018). Rationale, opportunities, and reality of biosimilar medications. N Engl J Med.

[20] Knight B, Rassam D, Liao S, Ewesuedo R (2016). A phase I pharmacokinetics study comparing PF-06439535 (a potential biosimilar) with bevacizumab in healthy male volunteers. Cancer Chemother Pharmacol.

[21] Hettema W, Wynne C, Lang B, Altendorfer M, Czeloth N, Lohmann R, Athalye S, Schliephake D (2017). A randomized, single-blind, Phase I trial (INVICTAN-1) assessing the bioequivalence and safety of BI 695502, a bevacizumab biosimilar candidate, in healthy subjects. Expert Opin Investig Drugs.

[22] Markus R, Chow V, Pan Z, Hanes V (2017). A phase I, randomized, single-dose study evaluating the pharmacokinetic equivalence of biosimilar ABP 215 and bevacizumab in healthy adult men. Cancer Chemother Pharmacol.

[23] Tajima N, Martinez A, Kobayashi F, He L, Dewland P (2017). A phase 1 study comparing the proposed biosimilar BS-503a with bevacizumab in healthy male volunteers. Pharmacol Res Perspect.

[24] Lu JF, Bruno R, Eppler S, Novotny W, Lum B, Gaudreault J (2008). Clinical pharmacokinetics of bevacizumab in patients with solid tumors. Cancer Chemother Pharmacol.

[25] Wynne C, Schwabe C, Batra SS, Lopez-Lazaro L, Kankanwadi S (2018). A comparative pharmacokinetic study of DRL_BZ, a candidate biosimilar of bevacizumab, with Avastin® (EU and US) in healthy male subjects. Br J Clin Pharmacol.

